# Editorial: Precision exploration of plant germplasm resources: breakthroughs and applications in telomere-to-telomere (T2T) genomics

**DOI:** 10.3389/fpls.2025.1573144

**Published:** 2025-03-10

**Authors:** Longxin Wang, Kai-Hua Jia

**Affiliations:** ^1^ School of Biological Science and Technology, University of Jinan, Jinan, China; ^2^ Institute of Crop Germplasm Resources, Shandong Academy of Agricultural Sciences, Jinan, China

**Keywords:** genome assembly, telomere-to-telomere, Hi-C, transposable elements, germplasm resources

The precision exploration of plant germplasm resources through telomere-to-telomere (T2T) genomics marks a transformative step in the field of plant genomics, opening unprecedented opportunities for the in-depth understanding of plant genetic diversity, adaptability, and evolution. The goal of this Research Topic is to highlight recent advancements in sequencing and assembly technologies that allow for the construction of high-quality, full-length T2T genomes, and to explore how these breakthroughs can facilitate the conservation and utilization of valuable plant germplasm resources. Achieving T2T completeness is critical in providing an exhaustive representation of chromosomes, capturing genetic information that was previously elusive and paving the way for comprehensive annotations. This extensive genetic map offers deeper insights into gene function, genome structure, and the genetic basis of plant traits, all of which are fundamental for improving agricultural practices and ensuring the sustainability of plant biodiversity.

As we continue to witness the rapidly evolving landscape of sequencing technologies, this Research Topic aims to foster research that explores the vast potential of T2T genomic data. We focus on how these genomic insights can enhance species conservation efforts, inform breeding programs, and provide invaluable information for genetic resource management. Additionally, we delve into the role of transposable elements in shaping plant genomes, examining their dynamic interactions with genome architecture and their contribution to the genetic robustness required for adaptation and evolution. By bringing together cutting-edge research on T2T genome assembly, transposable element dynamics, and applications in plant breeding and conservation, this Research Topic serves as a comprehensive resource for researchers aiming to harness plant genomics for both the advancement of agricultural biotechnology and the preservation of genetic diversity within plant species. Ultimately, our objective is to catalyze further advancements in plant genomics that will contribute to a more sustainable and resilient future for global agriculture.

In a study examining the *R-gene* repertoire in huanglongbing (HLB)-resistant wild Australian limes, 19 *R-genes* were identified and compared to those found in susceptible cultivated citrus species (Liu et al.). The objective was to pinpoint *R-genes* involved in resistance to HLB. Using a specialized annotation pipeline, the study revealed that more than 70% of *R-genes* in wild Australian limes were unannotated in their genomes, while cultivated species contained up to 104% more *R-genes*. These *R-genes* were primarily clustered on chromosomes 3, 5, and 7, with their distribution closely correlating with the species’ resistance to HLB. Phylogenetic analysis demonstrated that the *R-gene* sequences reflected evolutionary divergence between wild and cultivated species, with distinct selection patterns observed in resistant and tolerant varieties. These findings shed light on the genetic foundation of HLB resistance in wild Australian limes, underscoring their potential for breeding HLB-resistant citrus cultivars and enhancing genome-assisted breeding approaches.

Grain number per spike, a pivotal agronomic trait dictating wheat yield, lacks a comprehensive understanding of its underlying mechanism in Pubing3228. The study of Wang et al. identifies molecular markers and candidate regions associated with grain number per spike in the wheat variety Pubing3228 using genetic mapping techniques like site-specific amplified fragment sequencing (SLAF-seq) and bulked segregation analysis (BSA). A total of 187,489 SNPs were identified between parental lines, with 164,018 SNPs in mixed pools. Association analysis using SNP-index and extended distribution (ED) algorithms revealed 25 significant regions linked to the trait, containing 399 annotated genes, including three with non-synonymous mutations directly related to grain number per spike. These findings deepen our understanding of the genetic mechanisms controlling wheat yield traits. The molecular markers identified can be used for marker-assisted selection (MAS), supporting breeding efforts to develop higher-yielding wheat varieties. Further functional analysis of these genes will provide insights into the regulatory networks governing spike development and help optimize agricultural practices.

Li et al. conducted on *Castanea mollissima* (Chinese chestnut) aimed to explore its genome and the molecular mechanisms governing starch and sugar synthesis, specifically focusing on the pollen xenia effect. This research involved high-quality genome sequencing using Illumina, PacBio, and Hi-C technologies to assemble a reference genome and conduct comprehensive gene annotation. A total of 35,008 protein-coding genes were predicted, and insights into starch biosynthesis, including the roles of key enzymes like granule-bound starch synthase and soluble starch synthase, were revealed. The study also identified significant pollen xenia effects influencing starch accumulation and fruit quality, which are crucial for improving chestnut yield and flavor. By integrating genomic and transcriptomic data, the research lays the groundwork for future functional studies and molecular breeding strategies to enhance chestnut quality, particularly in terms of starch content and other vital economic traits.

*Nicotiana tabacum* is a crucial crop with high economic value and significant importance as a model organism in plant biology. Tong et al. reviewed the progress and future directions in sequencing the *N. tabacum* genome, highlighting advancements in sequencing technologies, including long-read sequencing, optical mapping, and Hi-C. These methods have significantly improved the quality of *N. tabacum* genome assemblies, resulting in recent high-quality chromosome-level assemblies with substantial increases in contig N50 sizes. New cultivars are also being sequenced. However, the paper outlines the need for further improvements, particularly in developing a tobacco pan-genome and a telomere-to-telomere (T2T) genome. Such advancements would deepen our understanding of genetic diversity and agronomic traits, facilitating precision breeding. By providing a comprehensive view of the genome’s structure and gene content, they are expected to improve functional genomics and breeding strategies. The article emphasizes that continued refinement of *N. tabacum* genome sequencing is essential for advancing agricultural productivity and genetic research.

This Research Topic highlights significant advancements in plant genomics, particularly through telomere-to-telomere (T2T) genomics, which provides comprehensive and high-quality genome assemblies. It explores how these technologies enhance our understanding of genetic diversity, gene function, and genome structure, contributing to plant breeding, conservation, and agricultural sustainability. Studies on HLB-resistant citrus, wheat yield traits, Chinese chestnut, and *Nicotiana tabacum* demonstrate the potential of T2T genomics to uncover genetic mechanisms and support precision breeding.

